# ProtozoaDB 2.0: A *Trypanosoma Brucei* Case Study

**DOI:** 10.3390/pathogens6030032

**Published:** 2017-07-20

**Authors:** Rodrigo Jardim, Diogo Tschoeke, Alberto M. R. Dávila

**Affiliations:** 1 Computational and Systems Biology Laboratory, Oswaldo Cruz Institute, Fiocruz, Rio de Janeiro 21040-900, Brazil; rodrigo_jardim@fiocruz.br; 2 Microbiology Laboratory, Rio de Janeiro Federal University, Rio de Janeiro 21941-901, Brazil; diogoat@gmail.com; 3 Nucleus in Ecology and Socio-Environmental Development of Macaé (NUPEM), Rio de Janeiro Federal University, Macaé, Rio de Janeiro 21941-901, Brazil

**Keywords:** protozoa, information extraction, *Trypanosoma brucei*, trypanosomatids, ProtozoaDB

## Abstract

Over the last decade new species of Protozoa have been sequenced and deposited in GenBank. Analyzing large amounts of genomic data, especially using Next Generation Sequencing (NGS), is not a trivial task, considering that researchers used to deal or focus their studies on few genes or gene families or even small genomes. To facilitate the information extraction process from genomic data, we developed a database system called ProtozoaDB that included five genomes of Protozoa in its first version. In the present study, we present a new version of ProtozoaDB called ProtozoaDB 2.0, now with the genomes of 22 pathogenic Protozoa. The system has been fully remodeled to allow for new tools and a more expanded view of data, and now includes a number of analyses such as: (i) similarities with other databases (model organisms, the Conserved Domains Database, and the Protein Data Bank); (ii) visualization of KEGG metabolic pathways; (iii) the protein structure from PDB; (iv) homology inferences; (v) the search for related publications in PubMed; (vi) superfamily classification; and (vii) phenotype inferences based on comparisons with model organisms. ProtozoaDB 2.0 supports RESTful Web Services to make data access easier. Those services were written in Ruby language using Ruby on Rails (RoR). This new version also allows a more detailed analysis of the object of study, as well as expanding the number of genomes and proteomes available to the scientific community. In our case study, a group of prenyltransferase proteinsalready described in the literature was found to be a good drug target for Trypanosomatids.

## 1. Introduction

Over the last decade new species of Protozoa were sequenced and deposited in GenBank [1,2,3,4]. The availability of the primary genome sequence is a good starting point for the community to contribute further analyses (e.g., identification and functional annotation of coding sequences as well as comparative genomics analysis) in order to infer new information on the biology of these organisms. Analyzing large amounts of data generated by genomics experiments, especially using Next Generation Sequencing (NGS), is not a trivial task. The ongoing NGS technology makes the sequencing of more and more eukaryote genomes a reality, giving rise to new paradigms (either for the development and improvement of semi-automatic analysis/annotation systems for this huge amount of data, or for an object-view concept where raw reads are the main, fixed object, and assemblies with their annotations take a role of dynamically changing and modifying views of the object [5]).

**Figure 1:**
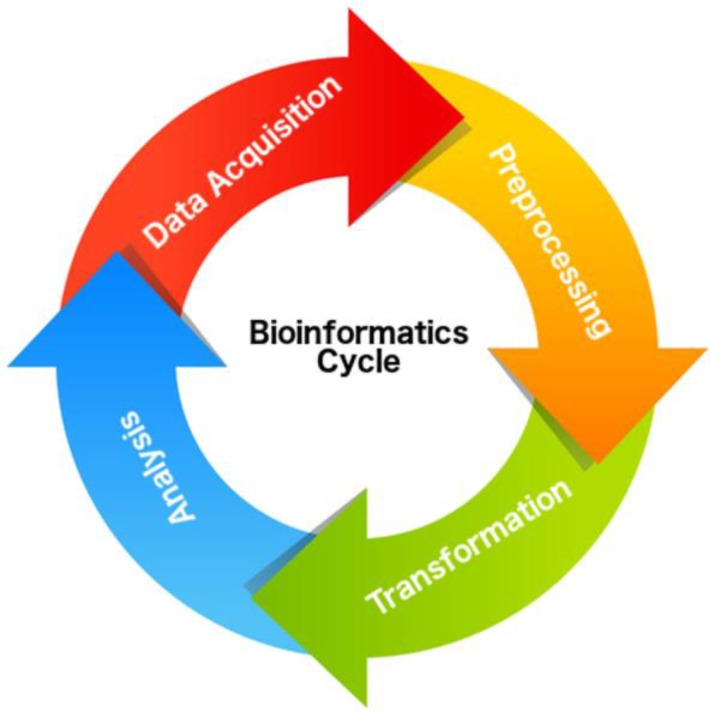
Example for data lifecycle in bioinformatics. The lifecycle begins with data acquisition, through data pre-processing, data transformation, and, finally, the analysis of the results (information) generated by this process.

The processes involved in the sequencing and preparation of genomic information can be represented in a similar way as the life cycle of software (Figure 1). The first step is data acquisition that can be performed by: (i) downloading from public databases; and (ii) sequencing across multiple platforms, like Sanger and/or NGS (Illumina, Ion Torrent, Nanopore and/or Pacific BioScience). The second step, called pre-processing, formats and stores genomic data for subsequent use. The third step refers to the use of a number of computational tools to transform raw data into knowledge. The fourth and last step is distributing and making this information available to the community for further analysis and inferences.

Therefore, in order to facilitate information extraction [6], we developed the ProtozoaDB [7] database system, which in its first version included five protozoan genomes (*Entamoeba histolytica*, *Leishmania major*, *Plasmodium falciparum*, *Trypanosoma cruzi*, and *T. brucei*) and a set of tools for searching and analyzing data, including phylogeny inference. In the present study we present a new version of ProtozoaDB called ProtozoaDB 2.0 (http://protozoadb.biowebdb.org) that, according to the above description, fits into the third and final steps of the bioinformatics cycle: transforming raw data into information followed by distribution and availability. The development of new generation databases as ProtozoaDB is being encouraged by the community, especially in the context of the BioCreative initiative [8] and reviewed by Krallinger et al. (2008) [9].

The system has been fully remodeled to allow for new tools and a more expanded view of data, using advanced computational techniques and providing a wider range of information for users. Now with the genomes of 22 pathogenic Protozoa, this new version includes analyses such as: (i) similarities with other databases (*Homo sapiens*, model organisms, Conserved Domains Database—CDD and Protein Data Bank—PDB); (ii) visualization of the metabolic pathways of Kyoto Encyclopedia of Genes and Genomes—KEGG [10]; (iii) protein structures by PDB [11]; (iv) homology studies, using results from OrthoMCL [12], KEGG Orthology (KO), and OrthoSearch [13]; (v) the search for related publications at PubMed; (vi) superfamily classification [14]; and (vii) phenotype inferences based on comparisons with model organisms, particularly with *Saccharomyces cerevisae*.

ProtozoaDB source code was completely rewritten in another programming language and with more elaborated techniques. It now uses a framework for developing Web applications known as Rails (http://rubyonrails.org/). It was developed in layers, allowing for the separation of the business object code of the pages displayed to users, making maintenance easier and consequently access to its pages lighter and faster. Furthermore, there is a specific layer to deal with data to be fetched from other sources. The Ruby language, suitable for the use of Rails, was adopted for this version together with BioRuby library [15], enabling the development of pages with less code and better reuse of functions. ProtozoaDB 2.0 was also implemented using concepts of Object Orientation and Design Patterns. This made the application lighter, safer, and simpler to maintain. The use of the JQuery library made possible for the web pages to work with Asynchronous Javascript and XML (AJAX), creating a friendlier user interface. Now it is possible to view all the information provided by ProtozoaDB 2.0 on one page. The new system uses the concept of Web Services to access all internal and external databases. Thus, the application focuses only on usability and user-friendly information. All databases are queried simultaneously allowing a response time considered to be satisfactory for the application. ProtozoaDB 2.0 allows queries by several methods, including: Genbank Identifier (GI), Accession Number, Description, Blast, Motif, and Phenotype. With all information in one place, it is now possible to infer information on the biology and biological systems of the protozoan species studied. Additionally, ProtozoaDB 2.0 now has information inferred from phenotypes. Orthology analysis helps to transfer phenotype information based on genotypes. According to [16] it is also possible to transfer functional information based on similar phenotypes and a specialized database called PhenomicDB was developed using this concept [17].

## 2. Results

### 2.1. Protozoa Genomic Data

**Figure 2:**
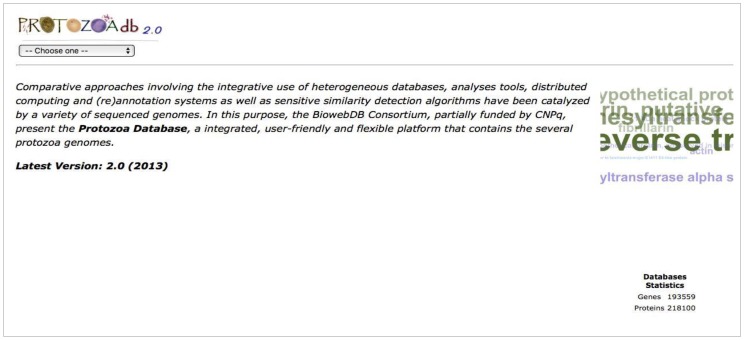
The front page of ProtozoaDB 2.0 displaying database statistics, the search field, and the tag’s cloud.

ProtozoaDB 2.0 provides descriptive, quantitative, qualitative, and comparative information on the genomes and proteins of 22 protozoan species (Table 1), thus allowing a more detailed analysis of each organism including the inference of relationships between them. The new version contains: (i) 193,559 genes; (ii) 218,100 proteins; (iii) 26,101 homologous groups (21,119 orthologous groups and 4982 paralogous groups) obtained by OrthoMCL analysis (Figure 2); and (iv) 195 phenotypes inferred by crossing information with the *Saccharomyces* Database.

### 2.2. Proteome

**Figure 3:**
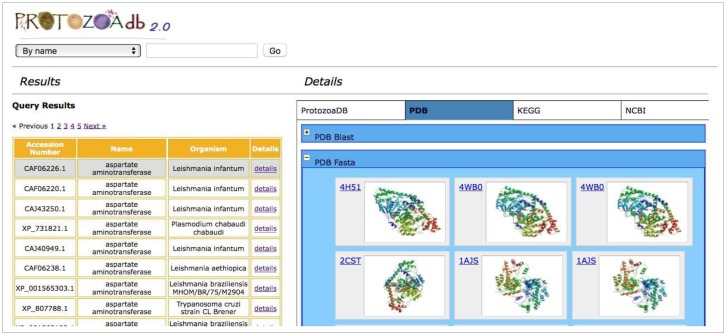
The similarity results against PDB: 2D (Blast) and 3D (Fasta). The figure shows sequence similarities displayed in 3D with the aspartate aminotransferase of *L. major*. Clicking on each figure of the system shows the complete information in the remote website.

**Figure 4:**
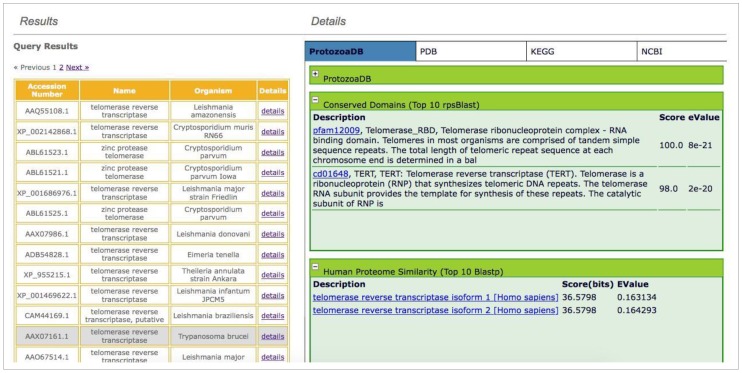
The results for similarity searches against *Homo sapiens* proteome and the Conserved Domains Database. Only the top ten results are shown. Clicking on links in blue opens a new window in the remote website.

The information about the proteins of 22 different Protozoa is complemented by the results obtained by real-time queries, performed in several remote databases through the use of Web Services. Two similarity analyses are performed using BLAST [18] and FASTA [19] against PDB [11]. The FASTA similarity results facilitate a visual comparison of the protein 3D structures, while the BLAST results also allow users to select any or all hits, as well as to retrieve and export their sequence in FASTA format (Figure 3). Conserved domains analyses use CDD [20]. As a plus, a similarity analysis against the human proteome is also performed. All this information is displayed showing the top 10 results (Figure 4).

### 2.3. Homology 

**Figure 5:**
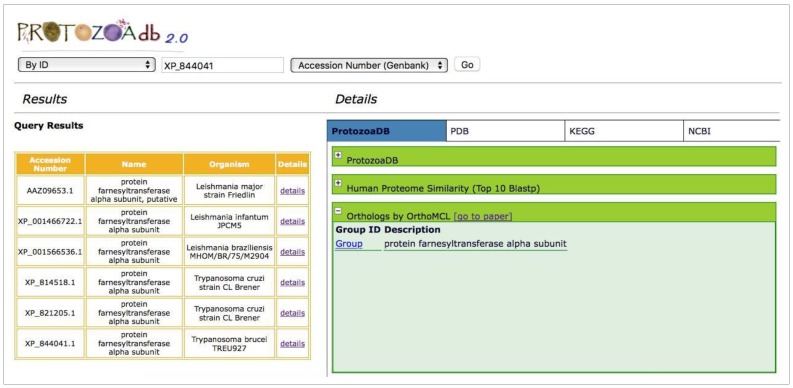
Orthologous groups inferred using OrthoMCL methodology. Clicking the “Group” link shows all proteins of that group that are shown in the left panel (Query Results).

The results of the preliminary analyses of homology among the 22 Protozoa are available for queries. The orthologous groups were inferred by the methodology implemented in OrthoMCL (Figure 5) and OrthoSearch, using either a Blast-based or Hmmer-based algorithm, respectively.

### 2.4. Metabolic Pathways

**Figure 6:**
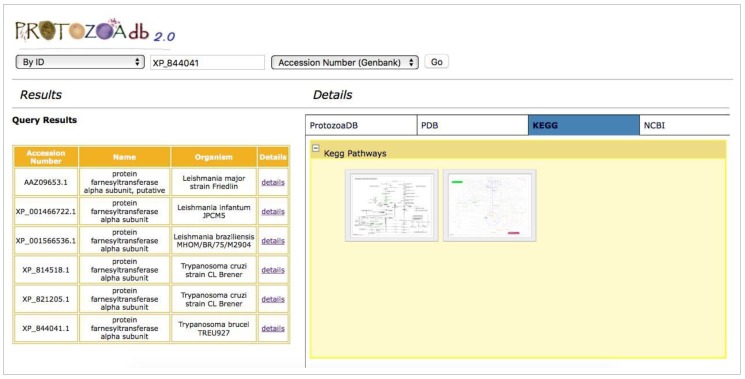
Metabolic pathways from KEGG. The figure shows all metabolic pathways that include aspartate aminotrasnferase. Clicking on a map opens a new window in a remote web site (KEGG).

The system performs a web service-based query to retrieve metabolic maps available on KEGG, showing the involvement of a given protein in that pathway (Figure 6).

### 2.5. Phenotypes

**Figure 7:**
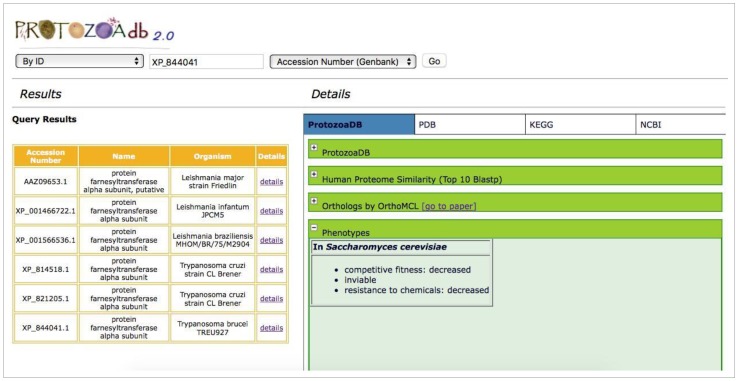
Phenotypes found by orthology with *Saccharomyces cerevisiae* for farnesyltransferase alpha protein subunit.

ProtozoaDB 2.0 allows web service-based queries through the phenotypes mapped from the *Saccharomyces* Database [21], retrieving proteins from the 22 Protozoa that could potentially provide such features. This information was made possible by mapping the proteome of the 22 species with information from the KEGG orthologous groups (Kegg Orthology—KO) as part of the “transformation” step described in the introduction (Figure 7).

### 2.6. How to Search

The new system retrieves the information through various search engines. Based on the previous version, the system searches for the description of the protein or part of the description, Accession Number, Genbank Identifier (GI), and organism name. In addition to these mechanisms, this new version also allows query by phenotype and similarity (Blast).

### 2.7. How to Search Using Our Web Service

In addition we also made a set of web service functions available to retrieve all information available in our system. The page http://services.biowebdb.org/howtouse contains the information about how to use available services including source code examples. Functions to search Protozoa proteins by Accession Number, Genbank Identifier, description (annotation), organism, phenotype, and Blast, as well as details of protein analyses like orthologous groups, similarity results, KEGG pathways, and phenotypes, are available for queries with our web services.

### 2.8. Information Extraction—T. brucei Case Study

**Figure 8:**
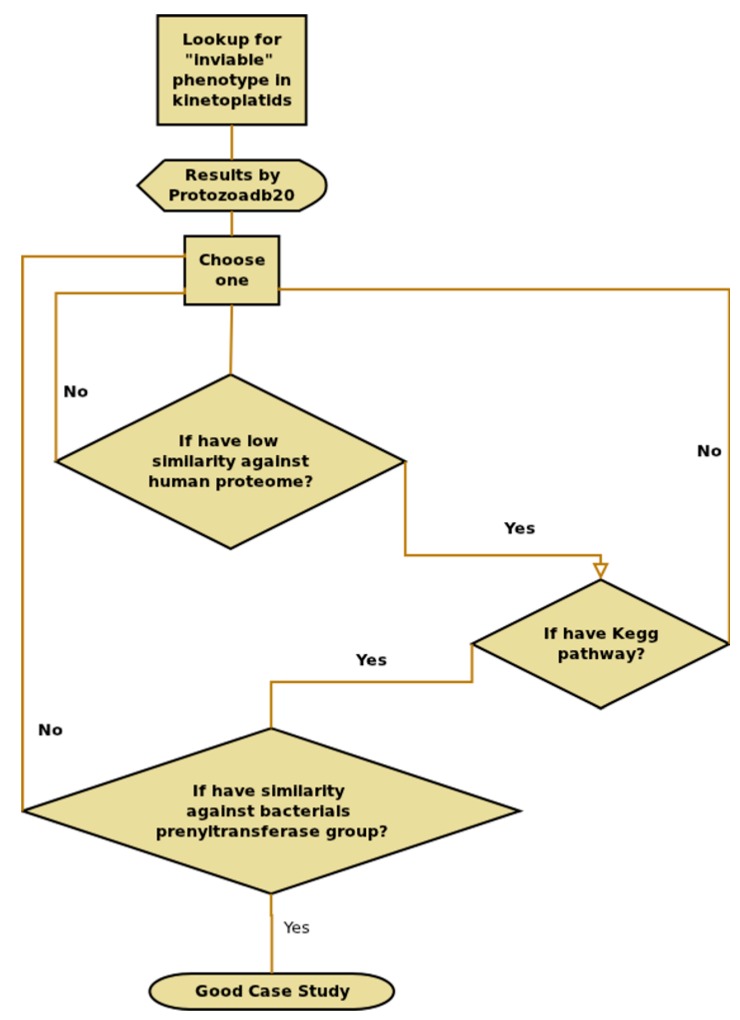
Flow chart showing the options and choices for the identification of a good case study.

To demonstrate the usefulness of ProtozoaDB 2.0 for information extraction, a case study was conducted using phenotypes in the Kinetoplastea species. Through the search field system the option Phenotypes was chosen and the keyword ”inviable” used with the Kinetoplastea subset database. This phenotype may indicate (depending on the experiment) a situation of impossibility for the survival of the organism [21]. Based on orthology with *Saccharomyces cerivisae*, the system returns a list containing a wide range of proteins that show this phenotype. From the obtained list, the first hit meeting the following requirements was chosen: (i) low similarity with the human proteome; (ii) high similarity with the bacterial species; and (iii) a pathway available in KEGG. The chosen hit was XP_844041.1 protein farnesyltransferase (PFT) alpha subunit from *Trypanosoma brucei*, because of the high similarity to the bacterial prenyltransferase group (Figure 8).

**Figure 9:**
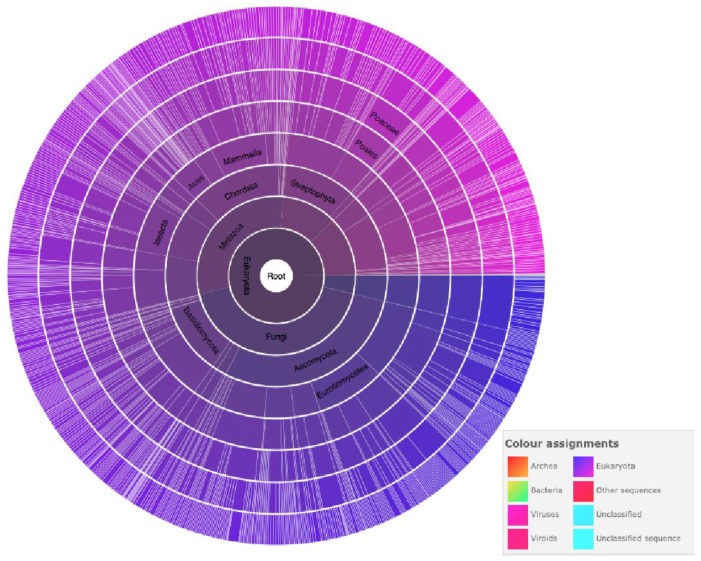
Distribution of prenyltransferase family across species, provided by PFAM. PFAM is part of EMBL-EBI and is provided as OpenScience [24].

Farnesyltransferase alpha subunit is a protein of the prenyltransferase group [22]. Pfam Farnesyltransferase and geranylgeranyltransferase are classified in the same family because of the CaaX motif present in both of them [23]. Figure 9 shows the PPTA family with 795 species of which 23% (223/795) are Metazoa that share this motif.

### 2.9. Comparison with Another Information Extraction Tool

We performed a comparison of ProtozoaDB with EuPathDB [25] to evaluate the similarities and differences between these two information extraction tools (Table 2).

## 3. Discussion

The previous version of ProtozoaDB contained only five pathogenic protozoa and some basic analyses. ProtozoaDB 2.0 increased over 17 protozoan species, totalizing 22 genomes and proteomes. New analyses were added in this new version, such as: homology analysis among the 22 organisms, using two different approaches; and phenotype inferences through orthology with the model organism. Furthermore, to allow for more comprehensive information about these organisms, several queries were performed in real time in third party (remote) sites, retrieving information about the proteome of organisms.

There are some other databases containing Protozoa species [25,26]; however, ProtozoaDB is the first database and web server that provides “all-in-one” information about comparative genomics of 22 species.

The use of web services allows for a flexible system that: (i) integrates a range of related information; (ii) has direct access to information in their original (remote) sources; and (iii) does not use local storage data from third parties (remote databases) that could imply their periodic update. These advantages allow our system to be always updated, since most of the information is queried directly in source databases through web services. The use of web services is already a practice in bioinformatics, since a number of research groups are using this technology, e.g., BioSWR [27] and BOWS [28].

Using an AJAX-based framework enables ProtozoaDB 2.0 to perform all queries through web services while simultaneously making the response time queries quite suitable for online analysis. AJAX framework is used for modern web sites, including those related to health [29,30].

The new search engines, particularly through BLAST, allow researchers to query the ProtozoaDB 2.0 data directly by the protein or gene of interest, viewing several pieces of information. Thus, it is possible to find a potential drug target by just browsing through the system and using all the information provided.

### 3.1. T. Brucei Case Study

Farnesyltransferase is one enzyme of the prenyltransferase group, which attaches a 15-carbon isoprenoid farnesyl group to proteins with CAAX motif: a four-amino acid sequence at the carboxyl terminus of a protein [31]. Farnesylation is a type of prenylation, a post-translational modification of proteins [32], which binds a isoprenyl group (15-carbon isoprenoid) to a cysteine residue. In other words, protein farnesylation involves protein farnesyltransferase (PFT) that catalyzes the attachment of the farnesyl group from farnesyl pyrophosphate (FPP) to cysteine SH of the C-terminal sequence motif CAAX, where C is cysteine and usually, but not always, an aliphatic residue. The terminal amino acid is determinant of farnesylation because FTase is preferentially active on protein substrates with CAAX [19]. This is an important process to mediate protein-protein interactions and protein-membrane interactions [31,33].

Prenylation (farnesylation) and subsequent modifications are essential for correct membrane targeting and cellular functioning of a number of proteins in eukaryotic cells such as Ras superfamily GTPases [34]. The farnesyltransferase enzyme is heterodimeric and has two subunits: alpha (α) and beta (β). The α subunit consists of a double layer paired alpha helices piled up in parallel, whichpartly enfold the beta subunit like a mantle.

As shown in Figure 9, prenyltransferase alpha subunit is present in various eukaryote species and several studies show that this protein is potentially a good drug target for trypanosomatids [33], especially because inhibitors have potent activity against cultured forms and are less toxic to mammalian cells than parasite cells. Besides that, PFT inhibitors have been developed as antimalarial agents [35].

### 3.2. Comparison between ProtozoaDB 2.0 and EupathDB

Both information extraction tools evaluated have several features that allow a wider analysis on the organism studied. EuPathDB allows a more comprehensive view of the characteristics of the protein investigated, whereas ProtozoaDB 2.0 focuses its analysis to infer and/or confirm the functional annotation of a given protein, based on its primary annotation deposited in Genbank. Furthermore, ProtozoaDB 2.0 also allows a view of the biological role played by the protein in biological systems, including information on related literature. Through ProtozoaDB 2.0 it is possible to re-annotate some of the proteins identified as “hypothetical” through similarity-based programs as well as SuperFamily-based classification. Finally, using the tools provided by ProtozoaDB 2.0, it is also possible to infer potential drug targets, as described in our case study.

## 4. Materials and Methods

### 4.1. Web Services

ProtozoaDB 2.0 supports RESTful (REpresentational State Transfer) [36] Web Services to make data access easier. Available in http://services.biowebdb.org/howtouse, these services were written in Ruby language using Ruby on Rails (RoR), allowing access to the information about proteome of Protozoa available in ProtozoaDB 2.0 web application.

### 4.2. New Source Code

The source code was rewritten in Ruby using Ruby on Rails, which allowed the development of three layers: view, with web pages based on Asynchronous JavaScript and XML (AJAX); model, with search algorithms in remote web services; and the controller, which is an interface between view and model.

### 4.3. Data Acquisition

The primary dataset of the genome and proteome of 22 Protozoa species (Table 1), including families *Babesiidae*, *Cryptosporidiidae*, *Entamoebidae*, *Hexamitidae*, *Plasmodium*, *Theileriidae*, *Trichomonadidae*, and *Trypanosomatidae*, was downloaded from Genbank [1] in Flat File format (GBFF).

### 4.4. Preprocessing

Primary data were stored locally in a server with a Database Manage Systems (DBMS) through the Genomics Unified Schema (GUS) version 3.5 [37] framework. The chosen DBMS was the PostgreSQL, version 8.4. PostgreSQL is a open source object-relational database system [38].

### 4.5. Transformation

Some analyses were performed to incorporate new information to the primary dataset. Inferences about homologies were performed on Protozoa data and the results locally stored, namely: (i) orthology inference using OrthoMCL [12]; (ii) OrthoSearch [13], which uses the Hidden Markov Model (HMM) through HMMER (version 3.0) and best reciprocal hits; and (iii) the BLAST-based similarity results.

Phenotypes were retrieved from the *Saccharomyces* Database [21] and stored locally. Mappings between (i) KEGG Orthology (KO), (ii) *Saccharomyces cerevisae* proteins, and (iii) Protozoa proteins were performed and stored locally, aiming to infer phenotypes in Protozoa.

### 4.6. Analysis

Web services technologies are used to access several databases worldwide to complement existing information (Table 3), among them:

(1) Similarity against the human proteome: the system performs a query, through the web service; the human proteome is locally stored in the database, updated every six months, and returns the top ten hits, independent of the score or e-value.
(2) Search for conserved domains: by running RPSBlast against the Conserved Domain Database (CDD) [39], the system returns the top ten results independent of the score or e-value.
(3) Superfamily classification: the Superfamily database [14,40] has structural, functional, and evolutionary information of proteins from different genomes, including Protozoa. The new system performs a query through the web service, retrieving graphical information on the classification of superfamily.
(4) Similar protein structure: the system retrieves information from 2D and 3D similarity by performing BLAST [18] and FASTA [19] directly in the Protein Data Bank (PDB) [41,42].
(5) Metabolic pathways: the system performs a query for the KEGG Pathway database [10] to retrieve the metabolic pathways where a given protein participates, showing the maps and their interactions with other proteins participating in that pathway.
(6) Literature: finally, the system performs two queries in Pubmed (http://www.ncbi.nlm.nih.gov/pubmed) to retrieve the original publication of the protein and other publications having relevance to the organism and the product.

## 5. Conclusions

ProtozoaDB 2.0 allows a more detailed analysis of the object of study, and expands the number of genomes and proteomes available to the scientific community. In our case study, a group of protein prenyltransferases was found by just browsing through the results provided by the web service-based tools, developed for this new version. This protein is already described in the literature as a good drug target for trypanosomatids for the following reasons: (i) its inhibitors have potent activity against cultured forms of these parasites and these inhibitors are more toxic against parasite cells than mammalian cells; (ii) for T. brucei PFT (TbPFT), the substrate specificities and inhibitor selectivity are distinct from mammalian PFT; and (iii) efforts of the pharmaceutical industry to develop small molecule inhibitors of mammalian PFTs for anti-cancer purposes creates an abundance of compounds that can be screened for selective activity against parasites. We were able to identify this potential drug target using only an “In Silico”-based strategy and the information available in public databases integrated was into ProtozoaDB 2.0.

## Figures and Tables

**Table 1:** List of organism species loaded in ProtozoaDB 2.0.

Organism/Strain
*Babesia bovis T2Bo*
*Crypstosporidium parvum Iowa II*
*Cryptosporidium hominis TU502*
*Cryptosporidium muris RN66*
*Entamoeba dispar SAW760*
*Entamoeba histolytica HM1:IMSS*
*Giardia lamblia ATCC 50803*
*Leishmania braziliensis MHOM BR 75 M2904*
*Leishmania infantum JPCM5*
*Leishmania major Friedlin*
*Plasmodium berghei ANKA*
*Plasmodium chabaudi chabaudi AS*
*Plasmodium falciparum 3D7*
*Plasmodium knowlesi strain H*
*Plasmodium vivax SaI 1*
*Plasmodium yoelii yoelii 17XNL*
*Theileria annulata Ankara*
*Theileria parva Muguga*
*Toxoplasma gondii ME49*
*Trichomonas vaginalis G3*
*Trypanosoma brucei treu927*
*Trypanosoma cruzi CL Brener*

**Table 2:** Comparison with another information extraction tool (n/a = not available functionalities).

Functionalities	ProtozoaDB 2.0	EuPathDB
Blast similarities against *Homo sapiens*	Available	n/a
Blast similarities against model organisms	Available	n/a
Blast similarities against protozoa species	Available	Available
Blast similarities against CDD	Available	n/a
Blast similarities against PDB	Available	n/a
Similarities against Intepro Domains	n/a	Available
KEGG metabolic pathways	Available	n/a
Protein structures by PDB	Available	n/a
Homology study: OrthoMCL	Available	Available
Homology study: KEGG orthologous	Available	n/a
Homology study: OrthoSearch	Available	n/a
Publications at PubMed	Available	n/a
Phenotype Search	Available	n/a
SNP Characteristics Search	n/a	Available
Genomic Position Search	n/a	Available

**Table 3:** Web services accessed.

Information	URL
ProtozoaDB	http://services.biowebdb.org/howtouse
PDB	http://www.rcsb.org/pdb/software/rest.do
Kegg	http://www.kegg.jp/kegg/docs/keggapi.html
Pubmed (NCBI)	http://www.ncbi.nlm.nih.gov/books/NBK55693/
Superfamily	http://supfam.cs.bris.ac.uk/SUPERFAMILY/web_services.html
